# Clinical evaluation of Suanzaoren decoction for sleep disturbance and cancer-related fatigue in patients with lung cancer receiving chemotherapy: A retrospective study

**DOI:** 10.1097/MD.0000000000046500

**Published:** 2026-01-30

**Authors:** Hua Yang, Ling Zhou, Yingli Huang, Ting Yang

**Affiliations:** a Department of Oncology, First Affiliated Hospital of Nanchang University, Nanchang, Jiangxi, China; b Department of Integrated Traditional Chinese and Western Medicine, Jiangxi Cancer Hospital, Nanchang, Jiangxi, China.

**Keywords:** cancer-related fatigue, chemotherapy patients, lung cancer, sleep quality, Suanzaoren decoction

## Abstract

This retrospective study investigated the potential effects of Suanzaoren decoction (SZRD) on sleep quality and cancer-related fatigue (CRF) in lung cancer patients undergoing chemotherapy. Clinical data were collected from 84 hospitalized patients with sleep disturbances between June 2021 and December 2022. According to treatment records, 38 patients received SZRD in addition to standard nursing care, while 37 patients received standard care alone. Sleep quality was assessed using the Pittsburgh Sleep Quality Index (PSQI), and fatigue was evaluated with the cancer fatigue scale. For patients with available records, objective sleep parameters were obtained from actigraphy monitoring. The baseline demographic and clinical characteristics were comparable between the 2 groups. Patients who received SZRD had significantly lower PSQI scores after treatment compared with the control group (*P* < .05). Fatigue scores were also markedly reduced in the SZRD group (*P* < .05). Actigraphy records indicated that total sleep duration and the proportion of deep sleep were higher in the SZRD group (*P* < .05), while other parameters showed no significant differences between groups (*P* > .05). No adverse reactions were recorded. These findings suggest that SZRD may be associated with improved subjective sleep quality and reduce CRF in lung cancer patients receiving chemotherapy, with potential benefits also reflected in some objective sleep parameters. However, given the retrospective design and limited sample size, further prospective, large-scale studies are warranted to confirm these results.

## 1. Introduction

Lung cancer is a prevalent malignant tumor in China.^[1]^ Indeed, lung cancer has the highest morbidity and mortality rates among all malignant tumors in China. Chemotherapy is a standard treatment for patients diagnosed with lung cancer^[2]^ but chemotherapy is limited by side effects, including gastrointestinal dysfunction, bone marrow suppression, negative emotions, stress, exhaustion, and sleep disturbances.^[3,4]^ Leak et al^[5]^ reported that >80% of lung cancer patients undergoing chemotherapy report exhaustion. Palesh et al^[6]^ found that cancer patients, especially patients with lung cancer, have the highest prevalence of sleep disturbances.

Patients with lung cancer experience severe sleep disturbances and exhaustion following chemotherapy that significantly impacts the quality of life. Lung cancer chemotherapy primarily necessitates dietary adjustments, physical activity, psychotherapy, and other pharmaceutical treatments.^[7]^ Oral, intravenous, and local chemotherapy are treatment options for lung cancer patients. Oral chemotherapy is the most frequently used chemotherapy formulation and has the most rapid onset of effect. Administering medications intravenously is a safe and efficient short-term treatment but it is important to carefully monitor the amount and duration to prevent adverse effects.^[8]^ Oral medications are safe and dependable but Traditional Chinese Medicine (TCM) can prevent unwanted reactions.^[9]^ Cancer patients attribute TCM syndromes to a lack of Qi (i.e., a lack of energy, feeling fatigued or dizzy, and decreased renal function of the lungs and spleen).

The Suanzaoren decoction (SZRD) has few side effects, excellent safety, and alleviates clinical symptoms in cancer patients.^[10]^ The SZRD is a well-known remedy for addressing fatigue and sleeplessness. The SZRD consists of Semen Ziziphi spinosae, Chuanxiong, Zhimu, Poria cocos, and licorice. The main active substances in the SZRD include cholesterol, isolignans, and polysaccharides based on an analysis of the ingredients. Research has demonstrated that cellulose, vitamin C, and flavonoids from Semen Ziziphi spinosae induces sedative, hypnotic, and tranquilizing effects, glycyrrhizin in licorice has immunomodulatory and anti-inflammatory effects, and the polysaccharides in Poria cocos have the potential to enhance immunity.^[10,11]^ Patients with lung cancer receiving chemotherapy experience a syndrome characterized by exhaustion, bad mood, pain, and sleep disorders, which may indicate the presence of depression.^[12]^ Pain, sleep disturbances, negative emotions, and cancer-related exhaustion have a reciprocal impact, significantly diminishing the quality of life among cancer patients.^[13]^ Studies have shown that the active medicinal ingredients,^[10,11]^ such as saponin, cellulose, vitamin C, flavonoids, glycyrrhizin, and polysaccharides in the SZRD, reduce anxiety by regulating glutamate-induced Ca[2]^+^ levels, γ-aminobutyric acid A receptor receptor expression, postsynaptic 5-hydroxytryptamine 1A receptor antagonist activity, and brain-gut peptide levels. The SZRD inhibits excessive release of anxiety-related neurotransmitters and improves sleep quality, which ameliorates the cancer-related fatigue (CRF) symptoms in cancer patients.^[14–16]^

Given the high prevalence of fatigue and sleep disturbances in lung cancer patients receiving chemotherapy, it is important to explore effective supportive strategies. Based on existing clinical records, the present study retrospectively analyzed the effects of SZRD on sleep quality and CRF in lung cancer patients undergoing chemotherapy, aiming to provide real-world evidence on its potential role as a complementary intervention.

## 2. Patients and methods

### 2.1. Ethical approval

The Ethics Committee of the First Affiliated Hospital of Nanchang University (IIT-2024-Lin-E-212) approved this retrospective study. All patient data were anonymized and handled in accordance with the Declaration of Helsinki to ensure confidentiality and privacy. A signed, written informed consent form was obtained from each patient.

### 2.2. Convenient sampling method

This retrospective study included 84 patients with primary lung cancer who received chemotherapy and were hospitalized in our institution between January 2021 and December 2021. Clinical data were collected from electronic medical records. Based on the treatment received, patients were divided into the SZRD group (n = 42) and the control group (n = 42).

### 2.3. Eligibility criteria

The inclusion criteria were as follows:

1. Patients with histopathologically confirmed primary lung cancer;
2. diagnosis of CRF accompanied by sleep disturbance;
3. diagnosis of Qi deficiency syndrome according to TCM criteria;
4. patients receiving chemotherapy for the first time with 6 planned treatment cycles;
5. age ≥ 18 years.

The exclusion criteria were as follows:

1. Patients with severe primary comorbidities, including heart disease (New York Heart Association functional class III–IV), central nervous system disorders (e.g., intracranial infection, cerebral hemorrhage, metabolic encephalopathy, intracranial tumors), or severe kidney disease (e.g., rapidly progressive nephritis, membranous nephropathy, polycystic kidney disease, lupus nephritis, or uremia);
2. patients with severe psychiatric disorders, such as schizophrenia, manic episodes, or bipolar disorder;
3. patients who had received sedative medications or other TCM-based interventions within the previous 3 months;
4. patients with other conditions known to affect sleep, such as obstructive sleep apnea or periodic limb movement disorder.

### 2.4. Diagnostic criteria

The diagnostic criteria for primary lung cancer followed the Guidelines of the Chinese Clinical Oncology Society for the Diagnosis and Treatment of Primary Lung Cancer (2018 edition).^[17]^ CRF was defined according to the diagnostic criteria proposed by the Fatigue Coalition,^[18]^ requiring ≥1 core symptom (persistent fatigue, decreased energy, disproportionate need for rest) and ≥5 additional symptoms (e.g., limb weakness, insomnia or hypersomnia, emotional distress, impaired concentration, short-term memory loss). Sleep disturbance was determined by a Pittsburgh Sleep Quality Index (PSQI) score ≥ 5 combined with patient complaints of sleep problems.^[19]^ Qi deficiency syndrome was assessed according to the Guiding Principles for Clinical Research of New Drugs of TCM,^[20]^ which requires the presence of at least 2 primary manifestations (shortness of breath, fatigue, mental weariness, weak pulse) and one secondary manifestation (spontaneous sweating, lethargy, weak tongue). These criteria were confirmed through physician evaluation of the medical records.

### 2.5. Intervention methodology

All patients included in this retrospective study received identical routine nursing care during chemotherapy, which consisted of health education regarding potential adverse reactions, symptomatic management of chemotherapy-related side effects, nutritional support, and psychological counseling. The content, frequency, and duration of psychological and nutritional interventions, as well as the management of chemotherapy-related symptoms and concomitant medications, were equivalent between the 2 groups according to nursing and medical records. Based on treatment documentation, patients were divided into 2 groups according to whether they received SZRD in addition to routine care. Patients in the SZRD group were recorded as having received oral SZRD (one dose in the morning and one in the evening) for approximately one month starting from the first day of chemotherapy. Patients in the control group received routine nursing care alone without SZRD. Clinical evaluations, including symptom assessments, questionnaire results, and sleep-related parameters, were retrospectively collected from medical records at baseline (prior to chemotherapy), during treatment, and after treatment, according to the availability of follow-up documentation.

### 2.6. Decoction preparation

The SZRD used in this study was a self-prepared TCM prescription. The main constituents were as follows: Semen Ziziphi Spinosae (stir-fried, 15 g), Glycyrrhiza uralensis (licorice, 3 g), Anemarrhena asphodeloides (anemarrhena, 6 g), Poria cocos (6 g), and Chuanxiong (Ligusticum chuanxiong, 6 g). The herbs were washed, placed in a clay pot, and boiled with 1200 to 1600 mL of water over high heat for 15 minutes. The heat was then reduced, and the mixture was simmered for 30 to 40 minutes until approximately 400 mL of decoction remained. After filtration, the liquid was divided into 2 equal portions (200 mL each). According to medical records, patients in the SZRD group took one portion in the morning and one in the evening at a warm temperature during the treatment period.

### 2.7. Evaluation tools

Clinical and demographic characteristics, including age, gender, work status, education level, marital status, monthly family income, clinical staging, and number of chemotherapy or radiotherapy cycles, were retrospectively collected from medical records using a structured information form. Sleep quality was assessed using the PSQI developed by Buysse et al^[21]^ the Chinese version of the PSQI has been widely applied in clinical research and has demonstrated high reliability and validity, with a test–retest reliability of 0.994, a split-half reliability coefficient of 0.824, and a Cronbach α coefficient of 0.845.^[22]^ Confirmatory factor analysis has also indicated satisfactory construct validity, with χ^2^/df = 4.83, RMSEA = 0.09, NNFI = 0.96, CFI = 0.98, and GFI = 0.97, and the correlation coefficient between the total PSQI score and insomnia severity index was 0.842.^[22]^ The Chinese version has further demonstrated a Cronbach α of 0.85 and retest reliability of 0.81, supporting its suitability for use in evaluating sleep quality in domestic populations.^[23]^ The PSQI consists of 18 items across 7 dimensions, each scored from 0 to 3, with a total score ranging from 0 to 21. Higher scores indicate poorer sleep quality. In this study, a total PSQI score >7 was used as the reference value for abnormal sleep quality in patients with advanced cancer. CRF was evaluated using the cancer fatigue scale (CFS), which was originally developed and validated in 307 cancer patients.^[24]^ The Chinese version of the CFS has demonstrated good reliability and validity, with internal consistency of 0.88 and test–retest reliability (*R* > 0.80). The CFS consists of 15 items divided into 3 domains: physical (5 items), emotional (5 items), and cognitive (5 items). Each item is scored on a 5-point scale, and higher scores represent greater fatigue severity. For descriptive purposes, fatigue severity was classified as mild (1–5), moderate (6–10), or severe (11–15).^[24,25]^

### 2.8. Data collection

Primary Outcomes: The primary outcomes were the PSQI and the CFS scores. These data were retrospectively collected from patient medical records. Assessments were documented at baseline (prior to chemotherapy), during treatment, and at follow-up, according to the availability of clinical records.

Secondary outcomes: Objective sleep status served as the secondary outcome. For patients with available records of actigraphy monitoring (Actiwatch 16, Mini Mitter Company, Inc., Bend), sleep-related parameters were retrospectively extracted. The recorded parameters included total sleep time, proportion of deep sleep, proportion of light sleep, proportion of rapid eye movement sleep, and number of awakenings. Data were collected based on the documentation available at baseline, during treatment, and after treatment.

### 2.9. Statistical analysis

Data were abstracted from medical records and double-entered in Excel; a third reviewer verified accuracy against source records. Analyses were performed in Statistical Package for the Social Sciences 24.0 (IBM, Armonk). Continuous variables are reported as mean ± SD or median (IQR) and compared using independent-samples *t* tests or Mann–Whitney *U* tests, as appropriate. Categorical variables are summarized as n (%) and compared using Pearson χ^2^ or Fisher exact tests. For the primary outcomes (PSQI and CFS), between-group differences in post-treatment scores were evaluated with ANCOVA adjusting for baseline values. Sensitivity analyses used multivariable linear regression additionally adjusting for age, sex, and clinical stage. Longitudinal changes were examined with repeated-measures analysis of variance; sphericity was tested with Mauchly test and Greenhouse–Geisser corrections applied when violated. Actigraphy-derived sleep parameters were analyzed in the same framework to assess group, time, and interaction effects.

As this was a retrospective study, the sample size was determined by the number of eligible patients meeting the inclusion criteria within the study period. A post hoc power analysis indicated that with 38 patients in the SZRD group and 37 in the control group, the study had over 80% power (α = 0.05) to detect a moderate effect size (Cohen *d* ≈ 0.6) for the primary outcomes (PSQI and CFS scores). Two-sided *P* < .05 was considered statistically significant.

## 3. Results

### 3.1. Baseline characteristics

Among the initially identified patients, some were excluded due to incomplete records or transfer to other institutions. After applying the inclusion and exclusion criteria, 37 patients were included in the control group and 38 patients in the SZRD group. In the control group, there were 30 males and 7 females, with a mean age of 48.09 ± 7.12 years, while in the SZRD group, there were 29 males and 9 females, with a mean age of 47.75 ± 7.69 years. No statistically significant differences were observed in baseline demographic or clinical characteristics between the 2 groups (*P* > .05). Baseline data are summarized in Table 1.

**Table 1:** Comparison of 2 sets of baseline data [example (%)].

Items	Classification	Study group (N1 = 38)	Control group (N2 = 37)	χ^2^	*P*
Gender	Male	29 (76.32)	30 (81.08)	0.398	.611
	Female	9 (23.68)	7 (18.92)		
Education	Primary School	17 (44.74)	13 (35.14)	0.396	.598
	Middle School	12 (31.58)	11 (29.73)		
	High School and Technical Secondary School	7 (18.42)	9 (24.32)		
	Junior College and Above	2 (5.26)	4 (10.81)		
Marital status	Married	35 (92.11)	34 (91.89)	0.481	.863
	Divorced or Widowed	3 (7.89)	3 (8.11)		
Work status	Employed	14 (37.84)	17 (45.95)	0.575	.169
	Unemployed or retired	24 (63.16)	20 (54.05)		
Monthly household income	≤3000	4 (10.53)	6 (16.22)	0.845	.231
	3001–4000	11 (28.95)	13 (35.14)		
	4001–5000	15 (39.47)	14 (37.83)		
	>5000	8 (21.05)	4 (10.81)		
Clinical stage	Stage I	2 (5.26)	4 (10.81)	0.611	.356
	Stage II	7 (18.42)	3 (8.11)		
	Stage III	17 (44.74)	16 (43.24)		
	Stage IV	12 (31.58)	14 (37.84)		

### 3.2. Comparison of sleep quality scores

The PSQI scores showed consistent improvement in the SZRD group compared to the control group. At week 2 of treatment, the mean total PSQI score was significantly lower in the SZRD group (7.65 ± 1.11) than in the control group (12.44 ± 1.38, *P* < .05). At week 4, the SZRD group also demonstrated significantly lower PSQI scores (6.07 ± 1.23 vs 13.26 ± 1.29, *P* < .05). Two weeks after treatment completion, the difference remained significant (5.97 ± 1.30 vs 12.85 ± 1.24, *P* < .05). Dimension-specific analyses yielded consistent results, as shown in Table 2.

**Table 2:** Comparison of the Pittsburgh Sleep Quality Index scores at different time points (x ± s, study group, N1 = 38; control group, N2 = 37).

Dimension	Group	Pretreatment	2nd wk during treatment	4th wk during treatment	2 wk post-treatment	Between-group effect		Time effect			Interaction effect	
						*F*	*P*	*F*	*P*	*F*		*P*
Sleep quality	Study	2.05 ± 0.62	1.82 ± 0.60	1.49 ± 0.54	1.14 ± 0.55	59.23	<.001	89.04	<.001		68.43	<.001
	Control	2.11 ± 0.59	2.22 ± 0.48	2.15 ± 0.50	2.37 ± 0.58							
Time to fall asleep	Study	1.87 ± 0.55	1.21 ± 0.38	0.91 ± 0.43	0.74 ± 0.40	66.14	.039	99.24	<.001		152.23	<.001
	Control	1.92 ± 0.54	1.90 ± 0.66	1.95 ± 0.59	1.89 ± 0.61							
Sleep duration	Study	1.41 ± 0.44	0.88 ± 0.52	0.79 ± 0.60	0.67 ± 0.55	30.06	<.001	10.24	<.001		81.06	<.001
	Control	1.28 ± 0.39	1.50 ± 0.47	1.56 ± 0.45	1.46 ± 0.42							
Sleep efficiency	Study	2.05 ± 0.69	1.37 ± 0.77	0.93 ± 0.66	1.08 ± 0.73	18.44	<.001	37.22	<.001		53.57	<.001
	Control	1.90 ± 0.73	1.85 ± 0.65	2.08 ± 0.56	1.94 ± 0.62							
Sleep disorder	Study	1.82 ± 0.37	1.12 ± 0.44	0.89 ± 0.39	0.78 ± 0.51	56.85	<.001	48.05	.017		50.56	<.001
	Control	1.95 ± 0.40	2.00 ± 0.52	2.11 ± 0.49	1.98 ± 0.43							
Hypnotic drugs	Study	0.99 ± 0.38	0.66 ± 0.49	0.61 ± 0.47	0.57 ± 50	29.71	<.001	4.09	<.001		17.85	<.001
	Control	0.87 ± 0.42	0.90 ± 0.44	0.97 ± 0.39	1.01 ± 0.43							
Daytime dysfunction	Study	2.33 ± 0.48	1.45 ± 0.55	1.17 ± 0.46	0.94 ± 0.52	56.21	<.001	82.46	<.001		62.62	<.001
	Control	2.27 ± 0.51	2.19 ± 0.47	1.94 ± 0.55	2.20 ± 0.39							
Total score	Study	11.99 ± 1.00	7.65 ± 1.11	6.07 ± 1.23	5.97 ± 1.30	264.59	<.001	213.62	<.001		287.73	<.001
	Control	12.07 ± 1.21	12.44 ± 1.38	13.26 ± 1.29	12.85 ± 1.24							

### 3.3. Sleep parameters based on actigraphy

For patients with available actigraphy data, the SZRD group demonstrated a longer total sleep duration and higher proportion of deep sleep compared with the control group (*P* < .05). Significant intergroup, time, and interaction effects were observed for these 2 parameters. The time effect of the deep sleep proportion was also significant between groups (*P* < .05). No significant differences were found between groups in other sleep-related indices, including light sleep, rapid eye movement sleep, and number of awakenings (*P* > .05, Table 3).

**Table 3:** Comparison of objective sleep indicators among groups (x ± s, study group, N1 = 38; control group, N2 = 37).

Items	Group	Pretreatment	2nd wk during treatment	4th wk during treatment	2 wk post-treatment	Between-group effect		Time effect		Interaction effect	
						*F*	*P*	*F*	*P*	*F*	*P*
Total sleep duration (min)	Study	352.4 ± 5.9	359.6 ± 4.2	370.7 ± 6.3	365. 9 ± 6.7	23.42	<.001	125.05	<.001	84.35	<.001
	Control	348.2 ± 6.3	350.5 ± 3.4	350.0 ± 5.3	349.2 ± 4.9						
Deep sleep proportion (%)	Study	26.78 ± 9.53	26.91 ± 7.09	28.87 ± 6.89	27.92 ± 7.21	4.07	.81	12.84	<.001	6.96	.66
	Control	27.01 ± 7.08	27.44 ± 8.94	26.99 ± 7.35	27.85 ± 6.83						
Light sleep proportion (%)	Study	60.68 ± 12.12	59.68 ± 10.96	55.86 ± 9.37	57.91 ± 12.12	75.37	.12	238.15	1.26	7.10	.26
	Control	59.16 ± 15.36	60.62 ± 16.14	60.68 ± 14.42	59.77 ± 9.30						
REM (rapid eye movement) proportion (%)	Study	45.16 ± 16.21	42.09 ± 14.45	47.25 ± 11.68	42.41 ± 7.99	357.25	1.00	106.26	.71	71.25	.76
	Control	44.77 ± 14.39	45.58 ± 16.43	46.78 ± 12.61	44.79 ± 9.87						
Deep sleep continuity (min)	Study	173.5 ± 7.72	180.52 ± 10.95	186.22 ± 13.48	185.56 ± 12.36	57.39	.73	55.78	.63	125.34	.95
	Control	178.2 ± 5.46	180.44 ± 9.34	182.56 ± 9.83	181.58 ± 11.35						
Number of awakenings (times)	Study	1.76 ± 1.45	1.45 ± 1.23	1.52 ± 1.08	1.60 ± 1.32	5.215	.02	8.62	.06	11.26	.08
	Control	1.66 ± 1.27	1.70 ± 1.11	1.75 ± 1.29	1.77 ± 1.38						

### 3.4. Comparison of fatigue scores before and after intervention

The CFS scores decreased more markedly in the SZRD group compared with the control group. The total fatigue score was significantly lower in the SZRD group, with significant intergroup, time, and interaction effects (*P* < .05, Table 4).

**Table 4:** Comparison of fatigue scores at different times between groups (x ± s, study group, N1 = 38; control group, N2 = 37).

Items	Group	Pretreatment	2nd wk during treatment	4th wk during treatment	2 wk post-treatment	Between-group effect		Time effect		Interaction effect	
						*F*	*P*	*F*	*P*	*F*	*P*
Physical fatigue	Study	15.87 ± 2.88	13.66 ± 2.45	10.57 ± 2.09	9.46 ± 2.35	37.46	<.001	40.24	<.001	33.18	<.001
	Control	16.25 ± 3.01	15.88 ± 2.96	17.54 ± 3.33	16.78 ± 3.14						
Emotional fatigue	Study	9.97 ± 1.96	7.91 ± 1.99	6.00 ± 1.87	5.78 ± 2.00	20.40	<.001	25.48	<.001	19.26	<.001
	Control	10.64 ± 2.14	10.55 ± 2.25	9.99 ± 2.36	11.03 ± 1.98						
Cognitive fatigue	Study	10.54 ± 2.74	8.45 ± 2.29	7.58 ± 2.33	6.89 ± 1.89	17.85	<.001	22.69	<.001	25.22	<.001
	Control	10.96 ± 2.95	11.22 ± 2.31	10.59 ± 2.02	10.68 ± 1.99						
Total score	Study	35.79 ± 3.81	30.74 ± 3.56	27.62 ± 2.97	25.77 ± 3.21	49.00	<.001	56.41	<.001	64.42	<.001
	Control	36.37 ± 4.63	36.48 ± 4.23	34.55 ± 3.96	35.78 ± 4.00						

### 3.5. Adverse events

No adverse events related to SZRD or routine care were reported in either group based on retrospective medical record review.

## 4. Discussion

The present retrospective analysis demonstrated that baseline demographic and clinical characteristics were comparable between the 2 groups. Patients who received SZRD in addition to routine care had significantly lower PSQI scores compared with those receiving routine care alone. The CFS scores were also reduced in the SZRD group, suggesting that SZRD may be associated with better sleep quality and reduced fatigue. Based on actigraphy records, only total sleep duration and deep sleep proportion improved significantly, while other parameters showed no statistical difference.

### 4.1. Effect of Suanzaoren decoction on sleep quality

In TCM, insomnia is attributed to internal imbalance, including asthenia, disharmony of Yin and Yang, and dysfunction of the zang-fu organs.^[26]^ Chemotherapy-related adverse reactions may further aggravate sleep disturbances in lung cancer patients.^[27]^ SZRD has long been applied in TCM to improve insomnia caused by Yin and blood deficiency and to calm the mind. Pharmacological studies have suggested that Anemarrhena asphodeloides and Ligusticum chuanxiong exert sedative and neuroregulatory effects.^[28]^ In this study, SZRD appeared to be associated with improved subjective sleep quality, consistent with prior findings.^[10,11,28]^

However, the objective monitoring data showed only modest benefits, with improvement in sleep duration but no clear effects on light sleep, rapid eye movement sleep, or frequency of awakenings. Several factors may explain this discrepancy. First, differences exist between subjective and objective assessment of insomnia, and subjective scales may better capture patients’ perceived improvement.^[26,27]^ Du et al^[28]^ reported that a modified SZRD improved subjective sleep indices without significantly altering polysomnography results, consistent with the present findings. Second, the treatment duration in this study was relatively short; longer or repeated courses of SZRD might yield greater benefits, which requires further confirmation. Wearable devices such as actigraphy are increasingly applied to evaluate sleep in cancer patients, offering noninvasive and convenient monitoring.^[29,30]^ In this analysis, actigraphy confirmed that total sleep duration was superior in the SZRD group, although other indices were not significantly different, highlighting the need for further methodological validation.

### 4.2. Effect of Suanzaoren decoction on cancer-related fatigue

Before treatment, patients in both groups experienced moderate fatigue, which gradually worsens during chemotherapy in most clinical contexts.^[31]^ In this study, the SZRD group showed a progressive reduction in fatigue severity over time, suggesting a potential association between SZRD use and reduced CRF severity. CRF is considered part of “asthenia” in TCM, consistent with the common manifestations of Qi deficiency such as shortness of breath and exhaustion.^[32]^ Classical TCM texts, including the Huangdi Neijing and Jingui Yaolue, describe the role of SZRD in nourishing the heart, calming the spirit, and alleviating insomnia and fatigue.^[33]^ Previous studies have reported that nearly half of chemotherapy patients develop sleep disturbances, with multifactorial influences including treatment regimen, psychological status, pain, and demographic characteristics.^[34]^ Evidence suggests that SZRD not only reduces fatigue but may also improve cognition impaired by sleep disturbance.^[35]^ Pharmacological and network pharmacology studies further support the multitarget actions of SZRD, including anti-inflammatory, neurotransmitter-regulating, and sleep-improving effects.^[11,32–34]^ These findings are in agreement with the present retrospective analysis.

### 4.3. Limitations of this study

This study has several limitations. First, its retrospective design may introduce selection bias, recall bias, and incomplete data capture. Second, the sample size was relatively small and the analysis was conducted at a single center, limiting the generalizability of the findings. Third, treatment duration was short and long-term outcomes could not be assessed. Finally, the absence of randomization or blinding means that potential confounding factors cannot be excluded. Future large-scale, multicenter, prospective studies are warranted to validate the observed associations.

## 5. Conclusion

This retrospective analysis suggests that SZRD may be associated with alleviation of CRF and improvements in sleep quality in lung cancer patients with Qi-deficiency syndrome undergoing chemotherapy. The findings also support its favorable safety profile. However, given the limitations of retrospective design and small sample size, these results should be interpreted with caution. Further large-scale, prospective, randomized controlled studies are required to confirm the efficacy and safety of SZRD in this patient population.

## Author contributions

**Conceptualization:** Hua Yang, Yingli Huang, Ting Yang.

**Data curation:** Hua Yang, Ling Zhou, Ting Yang.

**Formal analysis:** Hua Yang, Yingli Huang, Ting Yang.

**Funding acquisition:** Ting Yang.

**Investigation:** Ling Zhou, Yingli Huang, Ting Yang.

**Writing – original draft:** Ling Zhou, Yingli Huang, Ting Yang.

**Writing – review & editing:** Ling Zhou, Yingli Huang, Ting Yang.

## References

[1] Cao M Li H Sun D Chen W. Cancer burden of major cancers in China: a need for sustainable actions. Cancer Commun (Lond). 2020;40:205–10. 32359212 10.1002/cac2.12025 PMC7667573

[2] Zhu D Shi X Nicholas S Ma Y He P. Estimated annual prevalence, medical service utilization and direct costs of lung cancer in urban China. Cancer Med. 2021;10:2914–23. 33749141 10.1002/cam4.3845 PMC8026923

[3] Koshimoto S Yamazaki T Amano K. Psychosocial factors and the need for multidisciplinary support in nutrition counselling for cancer chemotherapy patients. Nutrients. 2023;15:2712. 37375616 10.3390/nu15122712 PMC10303182

[4] Muscaritoli M Arends J Bachmann P. ESPEN practical guideline: clinical nutrition in cancer. Clin Nutr. 2021;40:2898–913. 33946039 10.1016/j.clnu.2021.02.005

[5] Bryant AL Walon AL Phillips B. Cancer-related fatigue: scientific progress has been made in 40 years. Clin J Oncol Nurs. 2015;19:137–9. 25840376 10.1188/15.CJON.137-139 PMC4879585

[6] Palesh OG Roscoe JA Mustian KM. Prevalence, demographics, and psychological associations of sleep disruption in patients with cancer: university of rochester cancer center-community clinical oncology program. J Clin Oncol. 2010;28:292–8. 19933917 10.1200/JCO.2009.22.5011 PMC2815717

[7] Harorani M Davodabady F Farahani Z Hezave AK Rafiei F. The effect of Benson’s relaxation response on sleep quality and anorexia in cancer patients undergoing chemotherapy: a randomized controlled trial. Complement Ther Med. 2020;50:102344. 32444038 10.1016/j.ctim.2020.102344

[8] Laface C Laforgia M Molinari P. Hepatic arterial infusion of chemotherapy for advanced hepatobiliary cancers: state of the art. Cancers (Basel). 2021;13:3091. 34205656 10.3390/cancers13123091 PMC8234226

[9] Chen W Lin J Yang T. Yifei sanjie formula or placebo with anlotinib as second-line or above treatment for metastatic non-small-cell lung cancer: study protocol for a double-blind, placebo-controlled randomized pilot study. Integr Cancer Ther. 2023;22:15347354221151147. 36710490 10.1177/15347354221151147 PMC9893062

[10] Liu XX Ma YQ Wang YG Zhong FX Yin XP Zhang QM. Suanzaoren decoction for the treatment of chronic insomnia: a systematic review and meta-analysis. Eur Rev Med Pharmacol Sci. 2022;26:8523–33. 36459033 10.26355/eurrev_202211_30388

[11] Wang S Zhao Y Hu X. Exploring the mechanism of Suanzaoren decoction in treatment of insomnia based on network pharmacology and molecular docking. Front Pharmacol. 2023;14:1145532. 37670944 10.3389/fphar.2023.1145532 PMC10475534

[12] Strik H Cassel W Teepker M. Why do our cancer patients sleep so badly? Sleep disorders in cancer patients: a frequent symptom with multiple causes. Oncol Res Treat. 2021;44:469–75. 34350870 10.1159/000518108

[13] Lin Y Bailey DE Docherty SL. Distinct morning and evening fatigue profiles in gastrointestinal cancer during chemotherapy. BMJ Support Palliat Care. 2023;13:e373–81. 10.1136/bmjspcare-2021-002914 PMC8627530 34049967

[14] Yang S Leng X Liu J. Treatment of adult chronic insomnia and the effect of assisting benzodiazepine withdrawl with a combination of suanzaoren decoction and huanglian wendan decoction: a multicenter, prospective cohort study. J Nanjing Univ Tradit Chin Med. 2023;39:1224–31.

[15] Barkhordar Z Maleki M Khodadadi Z Wraith D Negahdari F. A Bayesian approach on the two-piece scale mixtures of normal homoscedastic nonlinear regression models. J Appl Stat. 2020;49:1305–22. 35707508 10.1080/02664763.2020.1854203 PMC9041943

[16] Kolaczyk ED Krivitsky PN. On the question of effective sample size in network modeling: an asymptotic inquiry. Stat Sci. 2015;30:184–98. 26424933 10.1214/14-STS502 PMC4584154

[17] Hou L Wang T Chen D; Multi-omics Classifier for Pulmonary Nodules (MISSION) Collaborative Group. Prognostic and predictive value of the newly proposed grading system of invasive pulmonary adenocarcinoma in Chinese patients: a retrospective multicohort study. Mod Pathol. 2022;35:749–56. 35013526 10.1038/s41379-021-00994-5

[18] Fabi A Bhargava R Fatigoni S; ESMO Guidelines Committee. Electronic address: clinicalguidelines@esmo.org. Cancer-related fatigue: ESMO clinical practice guidelines for diagnosis and treatment. Ann Oncol. 2020;31:713–23. 32173483 10.1016/j.annonc.2020.02.016

[19] Zhang Y Ren R Yang L. Patterns of polysomnography parameters in 27 neuropsychiatric diseases: an umbrella review. Psychol Med. 2023;53:4675–95. 36377491 10.1017/S0033291722001581

[20] Zhai X Wang X Wang L Xiu L Wang W Pang X. Treating different diseases with the same method – a Traditional Chinese Medicine concept analyzed for its biological basis. Front Pharmacol. 2020;11:946. 32670064 10.3389/fphar.2020.00946 PMC7332878

[21] Buysse DJ Reynolds CF 3rd, Monk TH Berman SR Kupfer DJ. The Pittsburgh Sleep Quality Index: a new instrument for psychiatric practice and research. Psychiatry Res. 1989;28:193–213. 2748771 10.1016/0165-1781(89)90047-4

[22] Lu TY Li Y Xia P Zhang GQ Wu DR. Reliability and validity analysis of the pittsburgh sleep quality index. Chongqing Med. 2014;000:260–3.

[23] Liu X Tang M Hu L. Reliability and validity of Pittsburgh Sleep Quality Index. Chin J Psychiatry. 1996;29:103107.

[24] Lee H Park EY Sung JH. Validation of the Korean Version of the cancer fatigue scale in patients with cancer. Healthcare (Basel). 2023;11:1796. 37372914 10.3390/healthcare11121796 PMC10298199

[25] Zhang F Ding Y Han LS. Reliability and validity of the Chinese version of the cancer fatigue scale. Chin Mental Health J. 2011;25:810813.

[26] Fan X Su Z Nie S. Efficacy and safety of Chinese herbal medicine Long Dan Xie Gan Tang in insomnia: a systematic review and meta-analysis. Medicine (Baltim). 2020;99:e19410. 10.1097/MD.0000000000019410 PMC7220054 32176067

[27] Gong L Xie JB Luo Y. Research progress of quality control for the seed of Ziziphus jujuba var. spinosa (Bunge) Hu ex H.F. Chow (Suan-Zao-Ren) and its proprietary Chinese medicines. J Ethnopharmacol. 2023;307:116204. 36720435 10.1016/j.jep.2023.116204

[28] Du Y Wang J Jiang L. Screening the components in multi-biological samples and the comparative pharmacokinetic study in healthy and depression model rats of Suan-Zao-Ren decoction combined with a network pharmacology. J Ethnopharmacol. 2024;319(Pt 3):117360. 37898440 10.1016/j.jep.2023.117360

[29] Pavic M Klaas V Theile G Kraft J Tröster G Guckenberger M. Feasibility and usability aspects of continuous remote monitoring of health status in palliative cancer patients using wearables. Oncology (Huntingt). 2020;98:386–95. 10.1159/000501433 31336377

[30] Spina MA Andrillon T Quin N Wiley JF Rajaratnam SMW Bei B. Does providing feedback and guidance on sleep perceptions using sleep wearables improve insomnia? Findings from “Novel Insomnia Treatment Experiment”: a randomized controlled trial. Sleep. 2023;46:zsad167. 37294865 10.1093/sleep/zsad167 PMC10485571

[31] Morse L Kober KM Viele C. Subgroups of patients undergoing chemotherapy with distinct cognitive fatigue and evening physical fatigue profiles. Support Care Cancer. 2021;29:7985–98. 34218321 10.1007/s00520-021-06410-7

[32] Zhao Y Wang S Li J. Effectiveness and safety of traditional Chinese medical therapy for cancer-related fatigue: a systematic review and Meta-analysis of randomized controlled trials. J Tradit Chin Med. 2020;40:738–48. 33000574 10.19852/j.cnki.jtcm.2020.05.003

[33] Huang HL Yang SB Mei ZG. Efficacy and safety of electroacupuncture combined with Suanzaoren decoction for insomnia following stroke: study protocol for a randomized controlled trial. Trials. 2021;22:485. 34496928 10.1186/s13063-021-05399-y PMC8427963

[34] Scher N Guetta L Draghi C. Sleep disorders and quality of life in patients with cancer: prospective observational study of the rafael institute. JMIR Form Res. 2022;6:e37371. 36422866 10.2196/37371 PMC9732755

[35] Cheng L Wang F Li ZH. Study on the active components and mechanism of Suanzaoren decoction in improving cognitive impairment caused by sleep deprivation. J Ethnopharmacol. 2022;296:115502. 35777606 10.1016/j.jep.2022.115502

